# Supplementary material for: Comparison between Gradual Reduced Nicotine Content and Usual Nicotine Content Groups on Subjective Cigarette Ratings in a Randomized Double-Blind Trial
Source: Int J Environ Res Public Health. 2020 Sep 26;17(19):7047. doi: 10.3390/ijerph17197047 (PMC7579069; doi:10.3390/ijerph17197047)
Supplement: Supplementary file 1 [file ijerph-17-07047-s001.pdf]

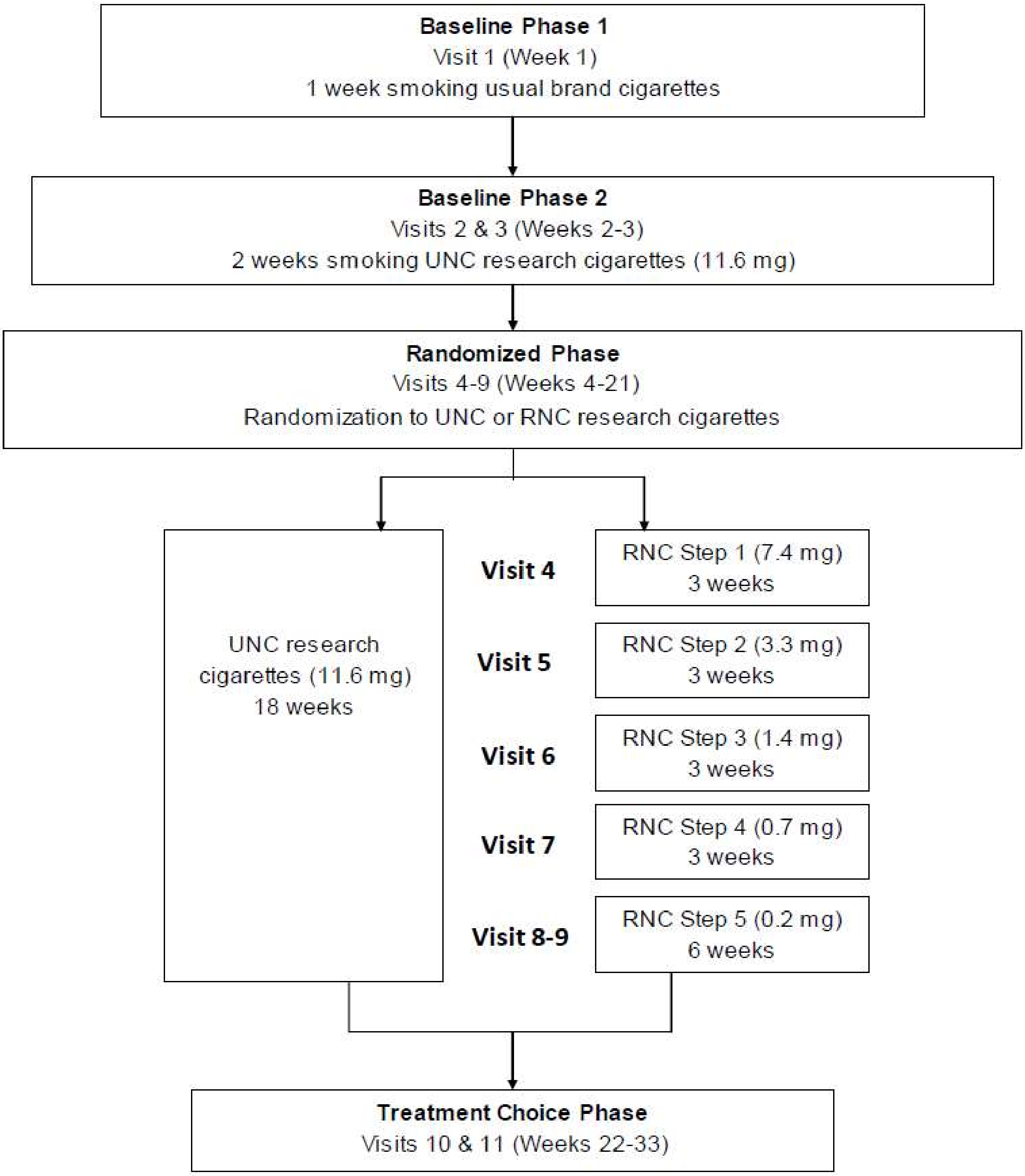

Figure S1. Study flow diagram.

Table S1. Demographic and smoking characteristics of randomized participants.

	Overall (N=245)	Usual Nicotine Content Group (N=123)	Reduced Nicotine Content Group (N=122)
**Baseline 1 Phase (Usual Brand Cigarettes)**			
[a]Age (years)	44.9 (11.4)	45.1 (10.8)	44.7 (12.1)
[a]Gender (male)	117 (47.8)	51 (41.5)	66 (54.1)
[a]Race (n=243)			
White	153 (62.4)	82 (67.2)	71 (58.7)
Black	78 (31.8)	35 (28.7)	43 (35.5)
Other	12 (4.8)	5 (4.1)	7 (5.7)
[a]Hispanic ethnicity	6 (2.4)	2 (1.6)	4 (3.3)
BMI[b] (kg/m^2)	30.3 (8.3)	30.8 (8.1)	29.9 (8.6)
[a]Education			
Less than a HS degree	40 (16.3)	25 (20.3)	15 (12.3)
HS degree or GED equivalent	112 (45.7)	57 (46.3)	55 (45.1)
More than a HS degree[c]	93 (38)	41 (33.3)	52 (42.6)
[a]Household income[d] (n=184)			
$0-19,999	68 (37)	30 (31.2)	38 (43.2)
$20,000-59,999	72 (39.1)	41 (42.7)	31 (35.2)
$60,000 +	44 (23.9)	25 (26)	19 (21.6)
[a]Employment status[e] (n=243)			
Currently working	108 (44.1)	54 (44.6)	54 (44.3)
Not currently working	135 (55.1)	67 (55.4)	68 (55.7)
Kessler Psychological Distress Scale score	4.1 (4.5)	4.2 (4.7)	4 (4.3)
Center for Epidemiologic Studies Depression Scale score	15.9 (7.1)	15.8 (7.3)	15.9 (6.9)
Monthly (or more) alcohol use in past year	66 (26.9)	31(25.2)	35(28.7)
Monthly (or more) illegal drug use in the past year	18 (7.3)	6 (4.9)	12 (9.8)
Cigarettes/day	19.6 (9.3)	19.8 (10.2)	19.5 (8.4)
Age started smoking daily (years)	17.1 (4.7)	16.8 (4)	17.4 (5.3)
Number of years smoking daily	27.8 (11.8)	28.3 (11.3)	27.2 (12.3)
Number of lifetime quit attempts			
0	79 (32.2)	45 (36.6)	34 (28.6)
1-3	116 (47.3)	55 (44.7)	61 (51.2)
4 or more	47 (19.2)	23 (18.7)	24 (20.2)
Menthol flavor preference	170 (69.4)	85 (69.1)	85 (69.7)

Environmental Tobacco Smoke Exposure score			
Little or no ETS	52 (21.2)	26 (21.1)	26 (21.3)
Moderate ETS	41 (16.7)	20 (16.3)	21 (17.2)
High ETS	152 (62)	77 (62.6)	75 (61.5)
Fagerström Test for Cigarette Dependence score	6.1 (2.1)	6.2 (2.1)	6.1 (2.1)
Hooked on Nicotine Checklist score	7.4 (2.3)	7.3 (2.3)	7.5 (2.2)
Penn State Cigarette Dependence Index score	13.5 (3.2)	13.6 (3.2)	13.4 (3.3)
Exhaled carbon monoxide[f]	30 (15)	30 (15)	29 (15)
Plasma Cotinine (ng/mL)[g]	274 (151)	266 (157)	282 (145)
Median (Range)	253 (3-812)	237 (3-812)	261 (24-730)
**Baseline 2 Phase (UNC Study Cigarettes)**			
Total cigarettes/day	24.1 (12.5)	24 (13.9)	24.2 (11)
Study cigarettes/day	24 (12.6)	23.8 (14)	24.1 (11)
Fagerström Test for Cigarette Dependence score	6.3 (2.1)	6.2 (2.2)	6.3 (2)
Hooked on Nicotine Checklist score	6.9 (2.4)	6.9 (2.5)	7 (2.3)
Penn State Cigarette Dependence Index score	13.4 (3.2)	13.5 (3.3)	13.3 (3.2)
Exhaled carbon monoxide[f]	31 (17)	31 (16)	32 (17)
Plasma Cotinine (ng/ml)[g]	261 (148)	260 (149)	262 (149)
Median (Range)	237 (11-797)	238 (11-797)	234 (26-779)

Continuous measures reported as mean (standard deviation); Categorical measures reported as frequency (column percent)

BMI= body mass index; ETS= environmental tobacco smoke; HS= high school; GED= general education diploma; SD= standard deviation

[a]Measure incorporated from PhenX Toolkit [37] version October 5, 2015.

[b]Calculated as (V2 Weight (lbs.) x 703) / (V1 Height (in.))^2

[c]Less than a Bachelor's degree required for inclusion

[d]Total Family (Household) Income includes the participant's income + income of all family members living in the participant's household (before taxes for the last calendar year)

[e]Currently Working includes working full-time or part-time, military; Not Currently Working includes temporarily laid off, sick leave, maternity leave, looking for work, unemployed, retired, non-working disabled (permanently or temporarily), keeping house, non-working student

[f]Reflects the average of two CO measurements if >1 CO was collected at the visit for safety monitoring

[g]Values < Limit of Quantification (LOQ = 4.3) were coded as 3 (LOQ/sqrt(2))

Table S2. Results of analyses comparing mCEQ subscales in RNC vs. UNC groups.

No. of Visit	Visit 4		Visit 5		Visit 6		Visit 7		Visit 8		Visit 9	
Nicotine Content	7.4 mg		3.3 mg		1.4 mg		0.7 mg		0.2 mg		0.2 mg	
	Mean difference (95% CI)	*p*	Mean difference (95% CI)	*p*	Mean difference (95% CI)	*p*	Mean difference (95% CI)	*p*	Mean difference (95% CI)	*p*	Mean difference (95% CI)	*p*
Satisfaction[1]	0.11 (-0.23,0.46)	0.5214	0.09 (-0.7,0.44)	0.6304	-0.44 (-0.8, -0.08)	**0.0172**	-0.4 (-0.78, -0.03)	**0.0356**	-0.47 (-0.86, -0.08)	**0.0181**	-0.62 (-1.02, -0.22)	**0.0026**
Psychological reward[2]	0.07 (-0.21,0.36)	0.6113	0.06 (-0.23,0.35)	0.6874	-0.26 (-0.56,0.03)	0.0809	-0.02 (-0.33,0.28)	0.8768	-0.24 (-0.56,0.07)	0.1326	-0.39 (-0.72, -0.06)	**0.0192**
Aversion[3]	0.03 (-0.17,0.23)	0.7556	0.03 (-0.18,0.23)	0.7991	0.02 (-0.19,0.23)	0.8210	0.1 (-0.12,0.32)	0.3690	-0.05 (-0.27,0.18)	0.6835	0.01 (-0.22,0.25)	0.9083
Enjoyment of Respiratory Tract Sensations[4]	0.07 (-0.33,0.47)	0.7142	0.14 (-0.27,0.55)	0.4995	-0.21 (-0.63,0.2)	0.3161	-0.44 (-0.87, -0.003)	**0.0486**	0.04 (-0.41,0.49)	0.8528	-0.15 (-0.61,0.31)	0.5261
Craving reduction[5]	-0.2 (-0.69,0.29)	0.4241	-0.16 (-0.66,0.35)	0.5367	-0.71 (-1.23, -0.19)	**0.0072**	-0.23 (-0.77,0.31)	0.4021	-0.59 (-1.14, -0.03)	**0.0388**	-0.19 (-0.76,0.39)	0.5260

Note: Mean difference of RNC vs. UNC (95% CI). Bolded *p* values indicate significance. Linear mixed-effect models adjusted for baseline (Visit 3) measure of the outcome, flavor, site, age group, education group, brand, gender, race, FTND score, cotinine, employment status. 1: mCEQ subscale 1; 2: mCEQ subscale 2; 3: mCEQ subscale 3; 4: mCEQ subscale 4; 5: mCEQ subscale 5. Unit for Nicotine Content: mg/cigarette.

Table S3. Results of analyses comparing CLS in RNC vs. UNC groups.

	Visit 4		Visit 5		Visit 6		Visit 7		Visit 8		Visit 9	
	Mean difference (95% CI)	*p*	Mean difference (95% CI)	*p*	Mean difference (95% CI)	*p*	Mean difference (95% CI)	*p*	Mean difference (95% CI)	*p*	Mean difference (95% CI)	*p*
CLS 1	-0.61 (-1.18, -0.03)	**0.0386**	-0.76 (-1.35, -0.17)	**0.0114**	-1.35 (-1.95, -0.75)	**<0.0001**	-0.88 (-1.51, -0.26)	**0.0055**	-1.19 (-1.84, -0.55)	**0.0003**	-1.29 (-1.95, -0.62)	**0.0002**
CLS 2	-0.21 (-0.79,0.36)	0.4658	-0.68 (-1.26, -0.09)	**0.0235**	-0.51 (-1.11,0.09)	0.0935	-0.26 (-0.88,0.36)	0.4080	-0.56 (-1.2,0.08)	0.0863	-0.66 (-1.32,0.004)	0.0516
CLS 3	-0.03 (-0.52,0.45)	0.9012	-0.34 (-0.83,0.16)	0.1868	-0.16 (-0.67,0.35)	0.5481	-0.17 (-0.7,0.36)	0.5340	-0.27 (-0.82,0.28)	0.3355	-0.35 (-0.92,0.22)	0.2267
CLS 4	-0.2 (-0.82,0.41)	0.5187	-0.68 (-1.31, -0.05)	**0.0343**	-0.58 (-1.23,0.06)	0.0777	0.14 (-0.53,0.81)	0.6848	-0.74 (-1.43, -0.04)	**0.0371**	-0.92 (-1.63, -0.2)	**0.0119**
CLS 5	-0.53 (-1.09,0.02)	0.0593	-0.72 (-1.29, -0.16)	**0.0125**	-1.39 (-1.97, -0.81)	**<0.0001**	-1.07 (-1.67, -0.47)	**0.0005**	-0.99 (-1.62, -0.37)	**0.0018**	-1.66 (-2.3, -1.01)	**<0.0001**
CLS 6	-0.24 (-0.82,0.35)	0.4326	-0.67 (-1.27, -0.07)	**0.0290**	-1.3 (-1.92, -0.68)	**<0.0001**	-0.42 (-1.06,0.22)	0.1938	-0.84 (-1.5, -0.17)	**0.0135**	-1.42 (-2.11, -0.74)	**<0.0001**
CLS 7	-0.13 (-0.64,0.38)	0.6193	-0.19 (-0.71,0.33)	0.4824	-0.78 (-1.31, -0.25)	**0.0042**	-0.38 (-0.93,0.17)	0.1763	-0.96 (-1.54, -0.39)	**0.0010**	-0.81 (-1.4, -0.22)	**0.0069**
CLS 8	-0.23 (-0.9,0.45)	0.5096	-0.45 (-1.14,0.23)	0.1962	-0.95 (-1.65, -0.25)	**0.0082**	-1.03 (-1.76, -0.31)	**0.0054**	-1.21 (-1.97, -0.46)	**0.0016**	-1.48 (-2.25, -0.7)	**0.0002**
CLS 9	-0.19 (-0.44,0.06)	0.1305	-0.25 (-0.51,0.005)	0.0545	-0.55 (-0.81, -0.28)	**<0.0001**	-0.37 (-0.64, -0.1)	**0.0080**	-0.7 (-0.98, -0.42)	**<0.0001**	-0.71 (-1, -0.42)	**<0.0001**
CLS 10	-0.03 (-0.28,0.22)	0.8300	-0.12 (-0.38,0.14)	0.3574	-0.33 (-0.6, -0.07)	**0.0130**	-0.26 (-0.53,0.01)	0.0621	-0.42 (-0.71, -0.14)	**0.0033**	-0.42 (-0.71, -0.12)	**0.0054**

Note: Mean difference of RNC vs. UNC (95% CI). Bolded *p* values indicate significance. Linear mixed-effects models adjusted for baseline (visit 3) measure of the outcome, flavor, site, age group, education group, brand, sex, race, FTCD score, cotinine, and employment status. CLS items: CLS item 1: How strong was the cigarette? (1 = not at all, 10 = extremely); CLS item 2: How hot was the cigarette? (1 = not at all, 10 = extremely); CLS item 3: How hard was it to draw? (1 = not at all, 10 = extremely); CLS item 4: How harsh was the cigarette? (1 = not at all,10 = extremely); CLS item 5: How much taste did you get from the cigarette? (1 = not at all, 10 = extremely); CLS item 6: How satisfying was the cigarette? (1 = not at all, 10 = extremely); CLS item 7: How much tobacco vs. 'just air' did you get from the cigarette? (1 = just air, 10 = just tobacco); CLS item 8: What is the likelihood that you would buy cigarettes like these? (1 = not at all, 10 = extremely); CLS item 9: How much nicotine do you think these cigarettes gave you compared to your usual cigarettes? (1 = much less, 5 = much more); CLS item 10: How satisfying was the hit these cigarettes gave you compared to your usual cigarettes? (1 = much less, 5 = much more).
